# Simulation and Experimental Study of Moderate Electric Field (MEF) Effects on Inactivation of *Listeria monocytogenes* and *Vibrio parahaemolyticus* in Surimi Paste

**DOI:** 10.3390/foods15101670

**Published:** 2026-05-11

**Authors:** Beom-Su Cho, Jin Hong Mok, Seohyeon Choi, Minji Kim, Ji-Young Yang, Eunsoo Kim

**Affiliations:** 1 Department of Food Science and Technology, Pukyong National University, 45 Yongso-ro Nam-gu, Busan 48513, Republic of Korea; jbs4644@gmail.com (B.-S.C.); jhmok1024@dgu.edu (J.H.M.); jyyang@pknu.ac.kr (J.-Y.Y.); 2 Department of Food Science and Biotechnology, Dongguk University-Seoul, 32 Dongguk-ro, Ilsandong-gu, Goyang 10326, Republic of Korea; hyeon1383@dgu.ac.kr; 3 Research Center for Marine Integrated Bionics Technology, Pukyong National University, Busan 48513, Republic of Korea; minjikim@pknu.ac.kr; 4 College of Pharmacy and Research Institute for Drug Development, Pusan National University, Busan 46241, Republic of Korea

**Keywords:** moderate electric field, microbial inactivation, quality-deteriorating enzymes, surimi paste, electrophoretic motion simulation

## Abstract

The present study evaluated the efficacy of moderate electric fields (MEFs) treatments against surimi, an intermediate seafood protein product, to enhance microbial safety and food quality at mild temperatures and electric field strength. The pathogens that have been associated with seafood, such as *Listeria monocytogenes* and *Vibrio parahaemolyticus*, were selected and investigated under varying conditions of applied MEF duty cycle (DC, 50 or 100% square-wave form with 20 kHz at 34 V/cm), temperature (20–60 °C), and treatment time (up to 10 min) against different surimi concentrations (10–20%). Microbial reductions in both *L. monocytogenes* and *V. parahaemolyticus* significantly increased with elevated temperature at higher duty cycle, and a maximum log reduction of 7.2 and 5.9 was achieved at 60 °C under both DC50% and 100% after 10 min, respectively. The potential MEF-induced inactivation of quality-deteriorating enzymes in fish products, including trimethylamine-N-oxide (TMAO) reductase, serine- and cysteine- proteases, was numerically evaluated based on enzyme-specific electrophoretic temperature rise. Overall, these findings highlight MEF as a promising hurdle technology for enhancing both microbial safety and enzyme control in marine-based protein products.

## 1. Introduction

Recently, to meet growing nutritional and sustainability demands on health-promoting foods, blue (also known as aquatic) foods—fish, invertebrates, algae and aquatic plants captured or cultured in freshwater and marine ecosystems—have emerged as a valuable source of high-quality nutrients [1,2]. Surimi is a high-protein fish-based ingredient produced by finely mincing and washing fish flesh to remove water-soluble components, thereby concentrating myofibrillar proteins, and it is widely used as a key intermediate in processed seafood products such as crab legs, crab sticks, and fish cakes [3]. In addition, by utilizing finely minced fish, surimi allows for the efficient use of species with low commercial value or limited direct consumption potential as valuable protein sources; consequently, its industrial efficiency and economic viability have driven the growing demand for surimi-based products [4].

During surimi processing, however, its susceptibility to microbial contamination can lead to quality deterioration and thereby poses significant risks to both food safety and product quality [5]. For example, fishery resources and their products are highly vulnerable to quality deterioration during storage and distribution. In particular, they can accumulate harmful biogenic amines such as histamine and putrescine, while also supporting the growth of pathogenic microorganisms, including *Listeria* spp. and *Vibrio* spp. These undesirable changes pose a serious public health risk, as consumption of contaminated seafood may lead to foodborne illnesses characterized by symptoms such as watery diarrhea, abdominal cramps, nausea, and fever [6,7,8]. Moreover, these microorganisms secrete a range of detrimental enzymes within seafood matrices, such as endogenous and microbial proteases (e.g., cathepsin, pepsin), and amine-producing enzymes, which collectively accelerate muscle proteolysis, induce slimy textures and off-flavors, ultimately leading to deterioration in product quality [9,10].

To effectively mitigate these risks, the implementation of rigorous and well-controlled technical strategies is imperative, particularly those targeting microbial inactivation and the suppression of enzymatic activity during storage and distribution. While conventional high-temperature sterilization has been widely applied to eliminate pathogens responsible for foodborne illnesses—thereby improving food safety and extending shelf life [11]—prolonged exposure to elevated temperatures can induce protein denaturation. This not only deteriorates the sensory and functional qualities of aquatic foods, but also leads to the loss of essential nutrients and structural integrity [12]. Furthermore, prolonged exposure of surimi to mild thermal conditions (50–60 °C) during processing induces the modori phenomenon. At this temperature range, endogenous heat-stable proteases, including cathepsins and alkaline proteases, retain significant catalytic activity and promote extensive proteolysis of myofibrillar proteins, particularly myosin heavy chain. This degradation impairs protein–protein interactions essential for gel network formation, resulting in weakening and eventual disintegration of the three-dimensional gel matrix [4,13].

To address these limitations, many different approaches in non- or milder thermal manners, such as high-pressure processing (HPP), cold plasma (CP), pulsed electric field (PEF), and ultraviolet (UV) irradiation, have been suggested for the past few decades, and most of them are still being developed and require further improvement for commercial-scale processing [14,15]. As another alternative approach, moderate electric fields (MEFs)—aiming at controlling electropermeabilization with minimum thermal effect— featured as relatively lower electric fields (1–1000 V/cm) than PEF, have been suggested for food quality control and food manufacturing. Thereby, MEF can reduce the voltage for effective inactivation from several kilovolts to only several volts, which dramatically reduces the energy consumption and equipment complexity [16]. These MEF-based techniques have been utilized for food safety of liquid food samples, such as apple cider, kale-blended juice, and peach puree, against inactivation of various microorganisms, such as *Escherichia coli* K12, and *Listeria* spp. at milder temperatures [17,18,19,20]. However, the application of MEFs to complex solid–liquid multiphase systems remains limited, and systematic studies on process optimization are scarce. In particular, optimization is governed by multiple interdependent operational parameters, including treatment duration, electric field strength, duty cycle, and temperature, all of which critically influence process efficiency and system behavior.

Despite extensive experimental investigations, the molecular mechanisms underlying electric field–induced alterations in food matrices and microbial constituents still remain insufficiently understood. Molecular simulation approaches provide atomistic-level insights into system responses, enabling detailed characterization of enzyme activity and structural rearrangements under varying electric field conditions. Consequently, both the identification of optimal processing parameters and the elucidation of underlying mechanisms necessitate simulation-based frameworks capable of capturing the coupled effects of multiple interacting variables.

In the present study, the MEFs were applied to surimi as a representative model food matrix to evaluate the inactivation kinetics of key foodborne pathogens, *Listeria monocytogenes* (Gram-positive) and *Vibrio parahaemolyticus* (Gram-negative). In parallel, molecular simulation approaches were employed to predict protein denaturation and enzyme inactivation as functions of MEF-induced physicochemical alterations. Collectively, the integration of experimental and computational methodologies aims to generate MEF-relevant datasets to inform the design and optimization of unit operations in continuous food processing systems.

## 2. Materials and Methods

### 2.1. Strains and Preparation of Pathogen Cocktail

The preparation of a mixed bacterial cocktail was carried out with modifications based on Kim et al.’s study [21]. *Listeria monocytogenes* KCTC13064 and *Vibrio parahaemolyticus* KCTC1471 strains were obtained from the Korean Collection for Type Cultures (KCTC, Jeongeup, Republic of Korea). Isolated colonies were prepared by culturing the strains in 10 mL tryptic soy broth (TSB; Difco Laboratories Inc., Detroit, MI, USA), and the cultures were stored at −70 °C in a cryoprotective medium composed of DMSO, TSB, and 10% glycerine in a 1:4:5 ratio. Bacterial strains were streaked onto tryptic soy agar (TSA), incubated at 37 °C for 24 h, and then stored at 5 °C.

Pure isolated colonies of *L. monocytogenes* and *V. parahaemolyticus* were inoculated into TSB using a loop and cultivated at 37 °C for 24 h. The cultured bacterial solutions were then centrifuged at 4000× *g* for 10 min at 4 °C, after which the supernatant fluid was removed. To concentrate the cell, 20% of the volume of the removed supernatant was replaced with sterile buffered peptone water (BPW). The concentrated bacterial solutions were then mixed in a 1:1 ratio to prepare the cocktail. The cocktail was inoculated into the surimi samples at a final concentration of 1% (*v*/*v*). The initial inoculum levels of the pathogen cocktail were determined to be 8.1 log CFU/mL for *L. monocytogenes* and 6.1 log CFU/mL for *V. parahaemolyticus*.

### 2.2. Sample Preparation: Surimi Paste

Surimi composed of Stichaeidae, selected as a representative surimi-type marine fish for microbial inactivation and gel-forming studies, was purchased from Marukin Sato Suisan Co., Ltd. (Sapporo, Japan). It was stored in an ultra-low temperature freezer at −70 °C (DFU-014L, GMS Inc., Dongducheon, Republic of Korea) and thawed by immersion at 4 °C for 3 h before each experiment. Surimi and sterilized distilled water were precisely weighed and homogenized using a hand blender (EMHB-A200WK, Yuyao Wellknown Electric Appliance Co., Ltd., Yuyao, China) with multidirectional movements for 3 min. All equipment used for paste preparation (hand blender, spatula, double-jacketed beaker, etc.) and solvents were sterilized prior to use. Weighing, grinding, and preparation of the solid suspension were performed in a clean bench.

### 2.3. MEF System

**Figure 1:**
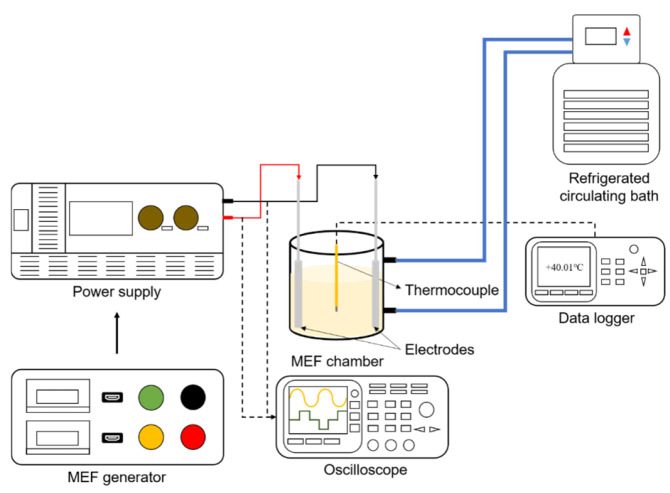
Schematic illustration of MEF setup. Electric pulses generated by the MEF signal generator are applied to the sample in a double-jacketed beaker, with real-time signal monitoring by an oscilloscope and commander system.

A double-jacketed beaker containing 100 g of surimi paste was equipped with fixed Pt–Ti plate electrodes (40 mm × 60 mm × 1 mm) with a 5 cm gap and placed on a magnetic stirrer operated at 1000 rpm, as shown in Figure 1. The differential probe (DP-25 Oscilloscope probe, Pintek Electronics Co., Ltd., New Taipei City, China) and a current probe (PT-2710, Pintek Electronics Co., Ltd.) were equipped to measure the electric current monitoring and recording. One end of a thermocouple (type K) was linked to the Data Acquisition System (DAQ970A, Keysight Technologies Inc., Santa Rosa, CA, USA), and the other end (temperature sensor) was placed at the center of the suspension for temperature monitoring and measurement. To prevent short circuits, the thermocouple, electrode, and magnetic bar were adjusted to avoid contact. To achieve and maintain the target sample temperature within the chamber, the outer jacket of a double-jacketed beaker was supplied with circulating cold water (5 °C) at controlled flow rates using a recirculating water bath (RW3-0535P, Jeio Tech Co., Ltd., Daejeon, Republic of Korea).

### 2.4. Pathogenic Microbial Inactivation

The microbiological analysis was carried out before and after the MEF treatments. A 100 μL sample was diluted stepwise with 900 μL of 0.85% NaCl solution. Each diluent was plated on tryptic soy agar (TSA) for *L. monocytogenes* and Thiosulfate-Citrate-Bile Salts-Sucrose (TCBS) agar for *V. parahaemolyticus*, followed by incubation at 37 °C for 48 h. Then the colonies formed on the plates were enumerated. Inactivation levels were expressed as log reduction ($N_{t}/N_{0}$), where $N_{t}$ represents the CFU/mL counts after *t* h of MEF, and $N_{0}$ refers to the initial value before treatment [22].

### 2.5. MEF Treatments

A series of experiments was conducted using 100 g of surimi suspension subjected to an electric field strength of 34 V/cm at 20 kHz, with various duty cycles (50% and 100%, leading to electric currents range between 0.9 and 1.3 A), temperatures (20, 40, and 60 °C), and surimi contents (10%, 15%, and 20% *w*/*w*). The operating parameters (34 V/cm at 20 kHz) were selected based on the operational limits of the MEF device. A maximum duty cycle of 100% was achievable only up to 20 kHz. Additionally, to enable stable adjustment and maintenance of target temperatures, including near-room temperature, under a fixed electrode distance, an electric field strength of 34 V/cm was applied. Inoculation with the mixed bacterial cocktail was performed after stable thermal equilibrium at the preset temperature was achieved and maintained constantly in the double-jacketed beaker. The moment at which the system reached thermal stabilization was defined as time zero for the treatment. Afterward, samples were collected at predetermined time points (1, 2, 5, and 10 min) to evaluate changes in microbiological properties over the course of the treatment. Thermal-only controls using conventional heating were performed under identical temperature and surimi content conditions without MEF. Sampling intervals and microbiological analyses were kept identical for direct comparability.

### 2.6. Mathematical Modeling of Inactivation Kinetics

Kinetic analysis on microbial inactivation was performed via D-values (the decimal reduction time that leads to a 10-fold reduction in surviving population) and parameters from the Weibull model using GraphPad Prism 10.4.2 for Windows (GraphPad Software Inc., San Diego, CA, USA). The Weibull model, based on the Weibull distribution, is given by:

(1) $$\log\frac{N}{N_{0}}=-{\left(\frac{t_{t}}{\delta }\right)}^{P}$$

In the Weibull equation, the parameter δ gives the time of the first decimal reduction. The shape of the curve is determined by the parameter P, ranging from concave (P < 1; so-called “tailing”) over linear (P = 1) to convex (P > 1; so-called “shoulder”) in a semi-logarithmic plot. The parameter $t_{t}$ indicates the treatment time. The goodness of the fit was assessed for each model, from the regression coefficient (R^2^), and the root mean squared error (RMSE) for the prediction of log ${N/N}_{0}$, where an RMSE closer to 0 indicates a better fit.

### 2.7. Molecular Dynamics Simulations

#### 2.7.1. Investigation of Enzyme Parameters Using the Database

Electrophoretic translational motion has been proposed as the mechanism behind nonthermal enzyme inactivation in electric fields, allowing the enzymes to behave as if subjected to a higher temperature than their surroundings. Two software platforms were used to determine enzyme parameters for kinetic simulations under an electric field. PDB2PQR 3.7.1 (Washington University in St. Louis, MO, USA) converts Protein Data Bank (PDB) files into PQR format, adding atomic charges and radii necessary for electrostatic calculations [23,24]. Adaptive Poisson-Boltzmann Solver (APBS) 3.4.1 (Pacific Northwest National Laboratory, Richland, WA, USA) computes biomolecular electrostatic properties by solving the Poisson–Boltzmann equation, enabling analysis of electrostatic interactions [25]. The parameters of each quality-degrading enzyme obtained through the above platforms are shown in Table 1.

#### 2.7.2. Numerical Modeling for Electrophoretic Motion of Enzymes in Surimi

The displacement of molecules induced by the electric field was numerically analyzed in MATLAB R2024a (The MathWorks, Inc., Natick, MA, USA) using ordinary differential equations. The modeling framework employed in this study included the following equations. Electrophoretic displacement ($s_{e}$) of the molecule:

(2) $$M\frac{d^{2}s_{e}}{dt^{2}}={q_{net}E}_{max}e^{j2\pi ft}-6\pi \eta r\frac{ds_{e}}{dt}$$

where M is molecular mass (kg), $q_{net}$ is the net molecular charge (C), $E_{max}$ is the peak value of the electric field strength (V/m), f is electric field frequency (=20,000 Hz), t is simulation time (=1/60 s), η is the viscosity of water, r is the radius of the molecule (m). Mean square displacement of a spherical particle due to Brownian motion, $<{S_{B}}^{2}>$:

(3) $$<{S_{B}}^{2}>=\frac{RT}{N_{A}}\frac{1}{3\pi \eta r}t$$

where R is universal gas constant (8.314 J/mol·K), T is temperature (K), *N_A_* is Avogadro’s number (6.022 × 10^23^). Total mean square displacement under MEF conditions, $<{S_{total}}^{2}>$:

(4) $$<{S_{total}}^{2}>=<{S_{B}}^{2}>+<{S_{e}}^{2}>$$

Effective temperature, induced by molecular displacement under MEF, $T_{eff}$:

(5) $$T_{eff}=\frac{3<{S_{total}}^{2}>N_{A}\pi \eta r}{Rt}$$

Electrophoretic temperature rise resulting from molecular motion under MEF, from which the final $∆T_{e}$ was derived:

(6) $$∆T_{e}=T_{eff}-T$$

A 3D graph was constructed with temperature on the *x*-axis, electric field strength on the *y*-axis, and effective temperature on the *z*-axis to predict the electrophoretically induced temperature rise in enzymes in response to variations in temperature and electric field intensity.

### 2.8. Statistical Analysis

All experiments were conducted in triplicate. Statistical analyses were performed using GraphPad Prism 10.4.2 for Windows (GraphPad Software Inc., San Diego, CA, USA). Data are represented as mean ± SD. Statistical analyses were performed using a two-way analysis of variance (ANOVA) with post hoc tests (Tukey) to assess statistical significance based on *p*-values (* *p* < 0.05; ** *p* < 0.01; *** *p* < 0.001; **** *p* < 0.0001).

## 3. Results

### 3.1. Microbial Inactivation and Related Kinetic Information

**Figure 2:**
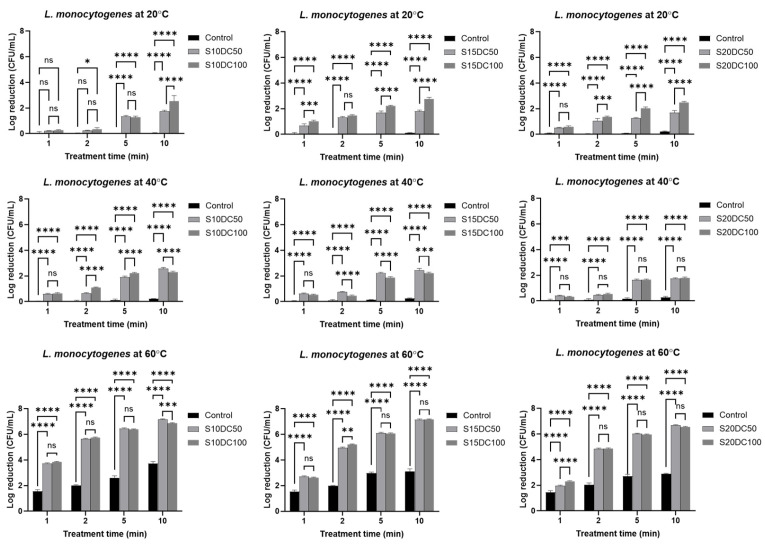
Reduction in bacteria bar charts corresponding to *L. monocytogenes* inactivation by different temperatures (20 °C, 40 °C, 60 °C), fish concentration (S10, S15, S20), and duty cycles (DC50, DC100) of MEF treatments (34 V/cm) for 10 min. Statistical significance was determined using a two-way ANOVA and Tukey’s post hoc test (* *p* < 0.05, ** *p* < 0.01, *** *p* < 0.001, **** *p* < 0.0001) for assessing the effect of duty cycle of MEF on *L. monocytogenes* inactivation; ns, not significant. Data shown as mean ± SD of three independent experiments.

**Figure 3:**
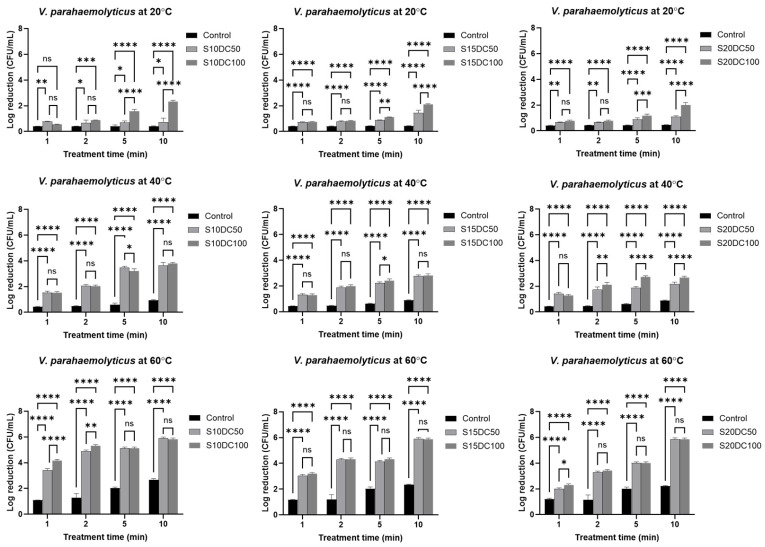
Reduction in bacteria bar charts corresponding to *V. parahaemolyticus* inactivation by different temperatures (20 °C, 40 °C, 60 °C), fish concentration (S10, S15, S20), and duty cycles (DC50, DC100) of MEF treatments (34 V/cm) for 10 min. Statistical significance was determined using a two-way ANOVA and Tukey’s post hoc test (* *p* < 0.05, ** *p* < 0.01, *** *p* < 0.001, **** *p* < 0.0001) for assessing the effect of duty cycle of MEF on *V. parahaemolyticus* inactivation; ns, not significant. Data shown as mean ± SD of three independent experiments.

In this study, the bactericidal effects against *L. monocytogenes* and *V. parahaemolyticus* were evaluated under varying temperatures and treatment durations with different surimi contents (Figure 2 and Figure 3, respectively). Experimental conditions were defined as follows: S10DC50 (10% surimi, MEF duty cycle of 50%), S10DC100, S15DC50, S15DC100, S20DC50, and S20DC100, and the corresponding log reductions were comparatively analyzed. For comparisons, inactivation of *L. monocytogenes* and *V. parahaemolyticus* by conventional heating and related kinetic parameters were included in the Supplementary Materials.

The inactivation of *L*. *monocytogenes* was quantified, and a maximum reduction of 7.2 log CFU/mL was observed at 60 °C. At 20 °C, microbial reductions were limited, up to 1.5 log reduction after 2 min and increasing up to 2.8 log reduction at 10 min, when it was treated with a duty cycle of 100%. Significant differences between treatment conditions were observed primarily at longer times, with DC of 100% achieving greater reductions (*p* < 0.05), while early treatment stages were mostly non-significant. At 40 °C, inactivation increased markedly, reaching 1.1 log reduction at the early treatment period (up to 2 min) and up to 2.6 log reduction at 10 min. Differences between DC of 50% and 100% were more evident at intermediate conditions (2–5 min) at lower surimi contents, suggesting that treatment intensity contributed substantially under moderate thermal conditions at higher moisture contents. At 60 °C, microbial reduction was most pronounced, achieving up to 3.8 log reduction after 1 min and exceeding 6.6 log CFU/mL reduction at 10 min. Under these conditions, most comparisons between the duty cycle of 50% and 100% were not statistically significant, indicating that thermal effects predominated over treatment variations. However, isolated significant differences observed at early time points suggest a minor contribution of treatment conditions, likely associated with variations in the thermal conductivity of the surimi paste suspension, which may influence electric current distribution within the solid–liquid matrix. MEF showed the greatest enhancement of *L*. *monocytogenes* inactivation at 60 °C after 10 min (up to 120% vs. thermal only, Figure 2 and Table S1).

The kinetic parameters from Table 2, including D-values and the Weibull model parameter (δ), demonstrate a strong temperature dependence of *L. monocytogenes* inactivation by applied MEF. At 20 °C, D-values ranged from approximately 4 to 7 min, whereas they decreased markedly to below 2 min at 60 °C, indicating accelerated microbial inactivation at the elevated temperatures. Consistently, the absolute slope values increased with temperature, reflecting an enhanced thermal contribution to lethality.

The reported thermal inactivation range for *L. monocytogenes* is typically 60–70 °C; for instance, D-values of 2.39 min in lobster meat and 2.61 min in crab meat at 60 °C have been documented [26]. In comparison, conventionally treated surimi at 60 °C exhibited D-values of 3.2–4.3 min (Table S2), whereas MEF-treated surimi paste showed markedly lower D-values (<1.9 min), indicating that the combined MEF and thermal treatment reduces microbial thermal resistance.

Modeling results indicated that the Weibull model provided a better fit (lower RMSE) for survival curves at lower temperatures, regardless of surimi content or MEF duty cycle. The majority of shape parameters (*P* < 1) exhibited upward concavity (tailing behavior), indicating that a more sensitive subpopulation of *L. monocytogenes* is rapidly inactivated, leaving behind a more resistant fraction [19,27]. Overall, the linear model showed comparatively better fitting performance at lower temperatures, whereas at higher temperatures neither the linear nor the Weibull models adequately described the inactivation kinetics under MEF conditions, suggesting increased complexity in microbial response due to the combined thermal–electric effects.

The inactivation effect of moderate electric field (MEF) treatment on *V. parahaemolyticus* was evaluated under the same conditions (Figure 3). Across all tested conditions, surimi pastes subjected to different duty cycles at low temperature after 10 min of treatment exhibited greater microbial reductions under the 100% duty cycle than under 50%. The effect of MEF on the inactivation of *V. parahaemolyticus* was greatest at 60 °C after 10 min, showing up to a 165% increase compared with thermal treatment alone (Figure 3 and Table S3).

The corresponding D-values (Table 3) decreased significantly with increasing temperature and duty cycle, indicating enhanced inactivation kinetics. Notably, results for the 100% duty cycle consistently yielded lower D-values and steeper survivor curve slopes than those for the 50% duty cycle, confirming the effectiveness of continuous current application in accelerating microbial inactivation. Additionally, parameters from the Weibull model showed low RMSE and high R^2^ values across all conditions, indicating a good model fit and reliable inactivation data.

At 40 °C, however, no significant differences were observed between different duty cycles of applied MEF in surimi paste containing 10% and 15% solids. In contrast, at 20% solid content, MEF treatment for 10 min resulted in 22.7% greater inactivation under a duty cycle of 100%, compared to that of 50%, indicating a synergistic effect between higher solid content and continuous electric current application.

In most results at 60 °C, no statistically significant differences in bactericidal efficacy were observed between duty cycles, suggesting that thermal effects predominated over electrical contributions. This observation is consistent with previous studies reporting that *V. parahaemolyticus* exhibits minimal inactivation at 20 °C, with an approximately 4-log reduction after 5 min at 60 °C under both cooking and microwave heating [28]. Only these conditions, the stronger thermal lethality up to 5.1 log reduction at 60 °C in low surimi solids, likely masked any additional effects of electric field modulation, derived from a more viscous sample.

In the kinetic results from *V. parahaemolyticus* inactivation under MEF, both linear and Weibull models demonstrated good agreement with the experimental data, as indicated by low RMSE along with high adjusted R^2^ values, with minor exceptions at 60 °C. Similar to results from *L. monocytogenes*, Weibull models with *P* < 1 exhibited upward concavity and tailing behavior, reflecting the rapid inactivation of susceptible *V. parahaemolyticus* populations followed by persistence of more thermally resistant cells. However, the Weibull model generally provided a better fit, with lower error metrics and higher R^2^ values. Therefore, the Weibull model was selected as the most appropriate for describing MEF inactivation kinetics of *V. parahaemolyticus* in surimi samples during milder thermal processing (<60 °C). In addition, similar trends were observed for the inactivation of *V. parahaemolyticus* in surimi samples by conventional heating (Table S4).

The enhanced microbial inactivation from both Gram-positive and Gram-negative bacteria observed under MEF treatment in the present study can be attributed primarily to electropermeabilization-mediated mechanisms. Exposure to MEFs induces alterations in the structural organization of membrane phospholipids and integral proteins, leading to the formation of transient or irreversible nanopores, modulation of ion channel activity, and a significant increase in membrane permeability [29]. These MEF-induced disruptions promote uncontrolled transmembrane transport of ions and intracellular constituents, resulting in dissipation of electrochemical gradients, osmotic imbalance, and loss of cellular homeostasis [30,31]. Collectively, these effects compromise membrane integrity and essential physiological functions, ultimately leading to irreversible cellular damage.

### 3.2. Inactivation of Quality-Deteriorating Enzyme Using Numerical Simulation

**Figure 4:**
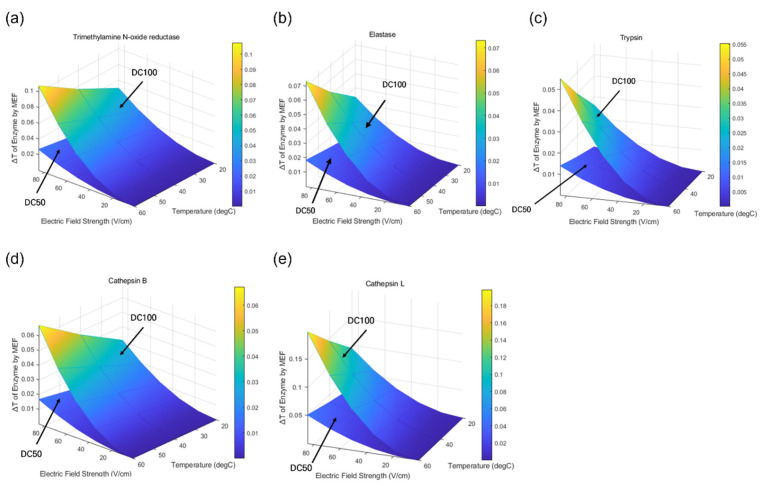
Estimated electrophoretic temperature rise ($∆T_{e}$) of quality-deteriorating enzymes under MEF (20–60 °C, 0–80 V/cm, 50 and 100% duty cycle). (**a**) TMAO reductase, (**b**) Elastase (serine protease), (**c**) Trypsin (serine protease), (**d**) Cathepsin B (cysteine protease), (**e**) Cathepsin L (cysteine protease).

To further elucidate these observations, numerical simulations were conducted to evaluate the electrophoretic displacement behavior of enzyme molecules under varying electric field strengths, temperatures, and duty cycles. Figure 4 illustrates the response surface analysis of the combined effects of electric field strength, temperature, and duty cycle on enzyme activity changes, along with electrophoretic temperature rise (ΔT) under MEF treatment at high frequency (20 kHz). The simulated results from the selected enzymes showed a sharp increase under conditions of elevated temperature, electric field strength, and duty cycle, which is consistent with the experimentally observed enhancement of nonthermal inactivation of tested microorganisms. This behavior can be attributed, in part, to intensified Brownian motion at higher temperatures, which increases molecular mobility and susceptibility to external electric forces.

Across all quality-deteriorating enzymes in surimi, ΔT increased with both electric field intensity and temperature, with consistently greater effects observed under continuous electric currents (duty cycle of 100%), compared to square-pulsed conditions (duty cycle of 50%). The nonlinear nature of the response surfaces indicates an accelerated rate of enzyme modification at higher processing intensities, suggesting a possible synergistic interaction between thermal and electrical factors on enzyme activity and stability.

Among the tested enzymes, the response of cathepsin L exhibited the highest sensitivity to MEF treatment, followed by trimethylamine N-oxide reductase, whereas trypsin, cathepsin B, and elastase were comparatively less affected. Overall, the electric field strength appeared to exert a more dominant influence than temperature, particularly at higher intensity ranges. These enzyme-specific structural characteristics play a critical role in determining their response to MEF. Variations in amino acid composition, hierarchical structural organization, molecular size, charge distribution, and conformational flexibility influence both the magnitude and distribution of MEF-induced forces. For example, trimethylamine N-oxide reductase, containing multiple domains and a metal prosthetic group, may experience localized electric field amplification, leading to destabilization of metal–ligand interactions and altered intramolecular energy dissipation. In contrast, serine proteases, such as elastase and trypsin, exhibit differential sensitivity due to variations in active-site flexibility and residue-specific thermal responsiveness. Similarly, cathepsin B and L, characterized by flexible loop regions and dynamic conformations, can become more prone to structural destabilization under combined thermal and electric stresses, potentially leading to enhanced loss of enzymatic activity.

This enzyme-specific simulation result may be associated with reduced turbidity at higher MEF heating rates, likely due to the shortened residence time near the myosin denaturation temperature. Furthermore, rapid heating limits the duration over which myosin remains within its denaturation range, thereby reducing the extent of protein aggregation [32]. Consistent with this observation, although MEF treatment at 60 °C may still induce partial protein denaturation, its volumetric heating by MEF significantly decreases the time required to reach and maintain denaturation temperatures. While our model simplifies enzymatic activity in an aqueous system without accounting for food matrix effects, this discrepancy should be acknowledged, as the microenvironment in concentrated protein gels, such as surimi, may differ substantially. In particular, matrix-induced changes in viscosity and electrical conductivity may alter electrophoretic motion and related activities. Nevertheless, the observed trends suggest that MEF treatment could help mitigate excessive protein aggregation and denaturation in surimi-based systems, highlighting its potential as a processing strategy for preserving protein quality. Overall, the effects of MEF on enzyme activity depend on both process parameters and intrinsic molecular properties, emphasizing the importance of enzyme-specific structural and physicochemical features for robust optimization of MEF-based food safety.

## 4. Conclusions

This study demonstrates that moderate electric field (MEF) treatment effectively targets both microbial hazards and quality-deteriorating enzymes in surimi paste samples. Experimental results confirmed significant inactivation of L. monocytogenes and V. parahaemolyticus, while simulations revealed the susceptibility of key enzymes, including trimethylamine N-oxide reductase, elastase, trypsin, cathepsin B, and cathepsin L. The inactivation efficacy was enhanced with increasing electric field strength and temperature, indicating a strong synergistic effect between electrical and thermal factors. Microbial inactivation was particularly pronounced at elevated temperatures, whereas enzyme destabilization was governed by both processing conditions and intrinsic structural properties, such as conformational flexibility and cofactor presence. Electrophoretic temperature rise, amplified by increased molecular mobility at higher temperatures, emerged as a critical factor driving enzyme inactivation. Collectively, these findings highlight MEF as a promising hurdle technology for simultaneous microbial control and enzyme inhibition. Future work should prioritize experimental validation of enzyme kinetics, process optimization for scale-up, and assessment of impacts on product quality to facilitate ‘blue food’ industrial application.

## Figures and Tables

**Table 1:** Values of parameters for molecular dynamics simulations of quality-deteriorating enzymes ^a^.

Database	3D Structure	*q_net_* (C)	*M* (kg)	*r* (m)	*η* (Pa·s)
TMAO (PDB ID: 1TMO)	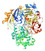	−1.122 × 10^−18^	1.474 × 10^−22^	4.443 × 10^−9^	1.002 × 10^−3^ (20 °C) 6.532 × 10^−4^ (40 °C) 4.665 × 10^−4^ (60 °C)
Elastase (PDB ID: 1EAI)	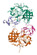	9.613 × 10^−19^	1.079 × 10^−22^	4.761 × 10^−9^	
Trypsin (PDB ID: 1UTJ)	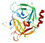	−4.806 × 10^−19^	3.957 × 10^−23^	1.556 × 10^−9^	
Cathepsin B (PDB ID: 1CP3)	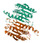	9.613 × 10^−18^	9.048 × 10^−23^	5.194 × 10^−9^	
Cathepsin L (PDB ID: 2YJC)	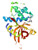	−1.282 × 10^−18^	3.990 × 10^−23^	3.075 × 10^−9^	

^a^ The values were obtained by PDB2PQR and APBS using the Protein Data Bank structures of enzymes.

**Table 2:** D-value and Weibull model parameters of *L. monocytogenes* inactivation in surimi paste samples subjected to MEF treatments.

Temp.	Sample	Kinetic Models						
		First-Order ^(a)^			Weibull Model ^(b)^			
		D-Value (min)	Slope	R^2^	*P*	δ	RMSE	R^2^
20 °C	S10DC50	5.4 ± 2.2	−0.186	0.918	0.27	11.78	0.099	0.931
	S10DC100	3.9 ± 0.3	−0.257	0.996	2.31	8.64	0.299	0.802
	S15DC50	6.5 ± 6.1	−0.154	0.674	0.12	6.17	0.174	0.858
	S15DC100	4.2 ± 2.5	−0.241	0.830	0.31	1.23	0.290	0.861
	S20DC50	6.9 ± 4.6	−0.146	0.802	0.17	30.96	0.232	0.659
	S20DC100	4.3 ± 2.5	−0.232	0.841	0.39	2.63	0.180	0.926
40 °C	S10DC50	3.9 ± 1.4	−0.255	0.939	0.32	1.61	0.127	0.966
	S10DC100	4.5 ± 3.0	−0.222	0.798	0.41	1.09	0.194	0.959
	S15DC50	4.1 ± 2.3	−0.245	0.853	0.20	1.14	0.070	0.986
	S15DC100	4.5 ± 2.2	−0.225	0.879	0.63	2.28	0.194	0.962
	S20DC50	5.6 ± 3.3	−0.180	0.836	0.62	3.45	0.192	0.938
	S20DC100	5.4 ± 3.0	−0.186	0.851	0.58	3.03	0.217	0.925
60 °C	S10DC50	1.8 ± 2.0	−0.554	0.606	0.51	0.28	0.558	0.949
	S10DC100	1.9 ± 2.3	−0.516	0.554	0.49	0.21	0.520	0.959
	S15DC50	1.7 ± 1.4	−0.603	0.723	0.52	0.29	0.455	0.965
	S15DC100	1.7 ± 1.5	−0.601	0.704	0.56	0.35	0.590	0.947
	S20DC50	1.7 ± 1.5	−0.587	0.703	0.54	0.35	0.553	0.946
	S20DC100	1.8 ± 1.6	−0.560	0.684	0.54	0.33	0.590	0.942

^(a)^ D-values are mean ± error propagation. Slope and R^2^ are from survivor curve regressions. ^(b)^ The δ is the scale parameter and *P* is the shape parameter of the Weibull model from microbial inactivation data. RMSE is the root mean square error.

**Table 3:** D-value and Weibull model parameters of *V. parahaemolyticus* inactivation in surimi paste samples subjected to MEF treatments.

Temp.	Sample	Kinetic Models						
		First-Order ^(a)^			Weibull Model ^(b)^			
		D-Value (min)	Slope	R^2^	*P*	δ	RMSE	R^2^
20 °C	S10DC50	5.7 ± 8.7	−0.175	0.904	0.33	0.19	0.333	0.946
	S10DC100	4.6 ± 5.1	−0.217	0.946	0.37	0.27	0.189	0.983
	S15DC50	8.9 ± 22.2	−0.113	0.776	0.28	0.26	0.121	0.987
	S15DC100	5.5 ± 8.0	−0.181	0.912	0.29	0.25	0.154	0.981
	S20DC50	11.9 ± 38.8	−0.084	0.671	0.17	0.11	0.100	0.987
	S20DC100	5.9 ± 9.0	−0.171	0.902	0.26	0.17	0.258	0.948
40 °C	S10DC50	3.1 ± 2.5	−0.320	0.740	0.34	0.22	0.299	0.956
	S10DC100	3.0 ± 2.0	−0.331	0.807	0.37	0.28	0.166	0.987
	S15DC50	4.5 ± 3.9	−0.221	0.709	0.28	0.28	0.123	0.986
	S15DC100	4.4 ± 4.0	−0.226	0.686	0.28	0.22	0.151	0.981
	S20DC50	6.5 ± 8.2	−0.154	0.532	0.20	0.19	0.102	0.986
	S20DC100	4.6 ± 5.0	−0.219	0.600	0.24	0.13	0.213	0.963
60 °C	S10DC50	2.3 ± 2.8	−0.432	0.555	0.58	0.53	0.187	0.992
	S10DC100	2.4 ± 3.1	−0.409	0.529	0.59	0.53	0.168	0.994
	S15DC50	2.3 ± 2.2	−0.442	0.660	0.61	0.65	0.191	0.991
	S15DC100	2.3 ± 2.3	−0.435	0.642	0.62	0.59	0.211	0.991
	S20DC50	2.0 ± 1.2	−0.499	0.845	0.62	0.70	0.194	0.991
	S20DC100	2.1 ± 1.3	−0.485	0.819	0.60	0.58	0.166	0.994

^(a)^ D-values are mean ± error propagation. Slope and R^2^ are from survivor curve regressions. ^(b)^ The δ is the scale parameter and *P* is the shape parameter of the Weibull model from microbial inactivation data. RMSE is the root mean square error.

## Data Availability

The original contributions presented in this study are included in the Article/Supplementary Materials. Further inquiries can be directed to the corresponding author.

## Supplementary Materials

The following supporting information can be downloaded at: https://www.mdpi.com/article/10.3390/foods15101670/s1, Table S1. Log reduction of *L. monocytogenes* in surimi paste samples subjected to conventional heating. Table S2. D-value and Weibull model parameters of *L. monocytogenes* inactivation in surimi paste samples subjected to conventional treatments. Table S3. Log reduction of *V. parahaemolyticus* in surimi paste samples subjected to conventional heating. Table S4. D-value and Weibull model parameters of *V. parahaemolyticus* inactivation in surimi paste samples subjected to conventional treatments.
