# Supplementary material for: Simulation and Experimental Study of Moderate Electric Field (MEF) Effects on Inactivation of Listeria monocytogenes and Vibrio parahaemolyticus in Surimi Paste
Source: Foods. 2026 May 11;15(10):1670. doi: 10.3390/foods15101670 (PMC13205550; doi:10.3390/foods15101670)
Supplement: Supplementary file 1 [file foods-15-01670-s001.zip › foods-4267560-supplementary.pdf]

foods

MDPI

Supplementary Materials

Simulation and Experimental Study of Moderate Electric Field (MEF) Effects on Inactivation of *Listeria monocytogenes* and *Vibrio parahaemolyticus* in Surimi Paste

Beom-Su Cho [1,†], Jin Hong Mok [1,2,†], Seohyun Choi [2], Minji Kim [3], Ji-Young Yang [1] and Eunsoo Kim [4,*]

[1] Department of Food Science and Technology, Pukyong National University, 45 Yongso-ro Nam-gu, Busan 48513, Republic of Korea; jbs4644@gmail.com (B.-S.C.); jhmok1024@dgu.edu (J.H.M.); jyyang@pknu.ac.kr (J.-Y.Y.)
[2] Department of Food Science and Biotechnology, Dongguk University-Seoul, 32, Dongguk-ro, Ilsandong-gu, Goyang-si 10326, Gyeonggi-do, Republic of Korea; 2021111658@dgu.ac.kr
[3] Research Center for Marine Integrated Bionics Technology, Pukyong National University, Busan 48513, Republic of Korea; minjikim@pknu.ac.kr
[4] College of Pharmacy and Research Institute for Drug Development, Pusan National University, Busan 46241, Republic of Korea
* Correspondence: eunsoo_kim@pusan.ac.kr
† These authors contributed equally to this work.

Table S1. Log reduction of *L. monocytogenes* in surimi paste samples subjected to conventional heating.

Temp.	Sample	Treatment time			
		**1 min**	**2 min**	**5 min**	**10 min**
20 °C	S10	0.07 ± 0.09	0.04 ± 0.02	0.03 ± 0.02	0.06 ± 0.02
	S15	0.04 ± 0.10	0.02 ± 0.02	0.02 ± 0.02	0.11 ± 0.02
	S20	0.08 ± 0.03	0.05 ± 0.01	0.08 ± 0.01	0.23 ± 0.04
40 °C	S10	0.02 ± 0.03	0.06 ± 0.05	0.12 ± 0.08	0.21 ± 0.02
	S15	0.04 ± 0.03	0.08 ± 0.08	0.14 ± 0.02	0.24 ± 0.05
	S20	0.06 ± 0.08	0.10 ± 0.08	0.16 ± 0.09	0.27 ± 0.09
60 °C	S10	1.55 ± 0.13	2.03 ± 0.04	2.61 ± 0.15	3.71 ± 0.17
	S15	1.55 ± 0.11	2.00 ± 0.02	2.67 ± 0.09	3.10 ± 0.21
	S20	1.46 ± 0.12	2.04 ± 0.14	2.71 ± 0.14	2.90 ± 0.04

Table S2. D-value and Weibull model parameters of *L. monocytogenes* inactivation in surimi paste samples subjected to conventional treatments.

Temp.	Sample	Kinetic models						
		**First-order**			**Weibull model**			
		**D-value (min)**	**Slope**	**R^2**	***P***	***δ***	**RMSE**	**R^2**
20°C	S10	108.3 ± 129.1	-0.009	0.560	3.104	21.18	0.047	0.445
	S15	75.6 ± 39.3	-0.013	0.870	1.742	32.24	0.049	0.554
	S20	51.5 ± 27.3	-0.019	0.865	1.977	23.40	0.063	0.613
40°C	S10	47.1 ± 5.5	-0.021	0.993	0.847	61.00	0.034	0.847
	S15	43.0 ± 7.6	-0.023	0.983	0.745	67.49	0.035	0.745
	S20	39.3 ± 9.5	-0.025	0.969	0.666	72.15	0.036	0.666
60°C	S10	3.2 ± 1.9	-0.309	0.829	0.383	0.35	0.136	0.990
	S15	4.0 ± 3.4	-0.251	0.712	0.292	0.19	0.103	0.993
	S20	4.3 ± 4.1	-0.235	0.658	0.270	0.16	0.167	0.980

[(a)] D-values are mean ± error propagation. Slope and R^2 are from survivor curve regressions. [(b)] The δ is the scale parameter and *P* is the shape parameter of the Weibull model from microbial inactivation data. RMSE is the root mean square error.

Table S3. Log reduction of *V. parahaemolyticus* in surimi paste samples subjected to conventional heating.

Temp.	Sample	Treatment time			
		1 min	2 min	5 min	10 min
20 °C	S10	0.40 ± 0.02	0.41 ± 0.02	0.41 ± 0.02	0.42 ± 0.02
	S15	0.41 ± 0.02	0.42 ± 0.02	0.43 ± 0.02	0.44 ± 0.02
	S20	0.43 ± 0.02	0.43 ± 0.02	0.45 ± 0.02	0.46 ± 0.02
40 °C	S10	0.44 ± 0.02	0.49 ± 0.02	0.59 ± 0.13	0.95 ± 0.05
	S15	0.43 ± 0.02	0.47 ± 0.02	0.63 ± 0.03	0.88 ± 0.04
	S20	0.42 ± 0.02	0.46 ± 0.01	0.59 ± 0.03	0.81 ± 0.04
60 °C	S10	1.09 ± 0.01	1.28 ± 0.33	2.06 ± 0.09	2.69 ± 0.09
	S15	1.19 ± 0.02	1.21 ± 0.36	2.04 ± 0.13	2.35 ± 0.04
	S20	1.21 ± 0.06	1.15 ± 0.38	2.00 ± 0.14	2.23 ± 0.04

Table S4. D-value and Weibull model parameters of *V. parahaemolyticus* inactivation in surimi paste samples subjected to conventional treatments.

Temp.	Sample	Kinetic models						
		First-order			Weibull model			
		D-value (min)	Slope	R^2	*P*	*δ*	RMSE	R^2
20 °C	S10	41.0 ± 86.7	-0.024	0.287	0.038	NR[c)]	0.009	0.998
	S15	38.9 ± 80.4	-0.026	0.296	0.045	NR	0.010	0.997
	S20	36.9 ± 74.5	-0.027	0.307	0.052	NR	0.012	0.997
40 °C	S10	13.2 ± 8.4	-0.076	0.814	0.371	25.28	0.086	0.938
	S15	14.1 ± 9.6	-0.071	0.795	0.343	21.50	0.040	0.984
	S20	15.5 ± 11.6	-0.064	0.765	0.314	31.43	0.038	0.984
60 °C	S10	3.7 ± 1.9	-0.274	0.864	0.613	3.39	0.365	0.907
	S15	4.2 ± 2.7	-0.237	0.818	0.546	3.64	0.361	0.886
	S20	4.4 ± 2.9	-0.225	0.808	0.533	3.84	0.357	0.879

[(a)] D-values are mean ± error propagation. Slope and R^2 are from survivor curve regressions. [(b)] The δ is the scale parameter and *P* is the shape parameter of the Weibull model from microbial inactivation data. RMSE is the root mean square error. [(c)] NR: not reliably estimated.
